# Medical Image Protection Algorithm Based on Deoxyribonucleic Acid Chain of Dynamic Length

**DOI:** 10.3389/fgene.2021.654663

**Published:** 2021-03-04

**Authors:** Xianglian Xue, Haiyan Jin, Dongsheng Zhou, Changjun Zhou

**Affiliations:** ^1^School of Computer Science and Engineering, Xi’an University of Technology, Xi’an, China; ^2^Sections of Computer Teaching and Research, Shaanxi University of Chinese Medicine, Xianyang, China; ^3^Shaanxi Key Laboratory for Network Computing and Security Technology, Xi’an University of Technology, Xi’an, China; ^4^Key Laboratory of Advanced Design and Intelligent Computing, Ministry of Education, Dalian University, Dalian, China; ^5^College of Mathematics and Computer Science, Zhejiang Normal University, Jinhua, China

**Keywords:** FOCHC system, DNA dynamic encoding, DNA dynamic chain, medical image encryption, deletion and transposition operation

## Abstract

Current image encryption algorithms have various deficiencies in effectively protecting medical images with large storage capacity and high pixel correlation. This article proposed a new image protection algorithm based on the deoxyribonucleic acid chain of dynamic length, which achieved image encryption by DNA dynamic coding, generation of DNA dynamic chain, and dynamic operation of row chain and column chain. First, the original image is encoded dynamically according to the binary bit from a pixel, and the DNA sequence matrix is scrambled. Second, DNA sequence matrices are dynamically segmented into DNA chains of different lengths. After that, row and column deletion operation and transposition operation of DNA dynamic chain are carried out, respectively, which made DNA chain matrix double shuffle. Finally, the encrypted image is got after recombining DNA chains of different lengths. The proposed algorithm was tested on a list of medical images. Results showed that the proposed algorithm showed excellent security performance, and it is immune to noise attack, occlusion attack, and all common cryptographic attacks.

## Introduction

Nowadays, technologies such as telemedicine, tele-surgery, and tele-radiology have been enormously developed and are in the preparation stage for clinical usage (Priyanka and Maheshkar, 2017). Patient information may be exposed to network transmission with these technologies. Especially, medical images (MRI, CT, and X-ray) with large data storage, redundancy and high pixel correlation are easily attacked and tampered by unauthorized access. Therefore, it is necessary to develop efficient high-performance medical image encryption method.

Since the ground-breaking work on DNA computing conducted and reported by Adleman (1994). DNA computing has attracted ever increasing attention of researchers worldwide, due to its superior characteristics of large concurrency, mass storage and low energy consumption (Li et al., 2020; Liu et al., 2020; Wang B. et al., 2020; Zhu et al., 2020). In 2009, DNA coding theory was used in the field of image information security by Zhang et al. (Xue et al., 2010a,b; Zhang et al., 2010; Liu et al., 2012; Zhang and Wei, 2013), which opened a new window for the DNA cryptography. The main encryption ideas were using the DNA operations (addition, subtraction, XOR, and DNA complement operations) and combination with some chaotic systems to achieve image encryption. Their novel methods and better encryption effects were often emulated and affirmed by researchers. However, Zhang et al.’s method was criticized as being unsafe in recent years. For instance, Zhu et al. (2017) and Hermassi et al. (2014) pointed out that the DNA addition operation proposed by Zhang et al. (2010) was irreversible. Besides, the encryption algorithm proposed by Zhang et al. (2012) has been deciphered by Belazi et al. (2014), Liu et al. (2014), and Wang et al. (2015) with chosen plaintext attack (CPA), respectively.

To improve the security, some researchers combined the complex chaotic systems with the DNA coding. For instance, Mondal and Mandal (2017) used two Logistic chaotic systems, and Zhang Y.Q. et al. (2016) used MLNCML system embedded logistic, to strengthen the existing algorithm. All of them combined the chaotic system with the DNA coding operations (addition and subtraction) to encrypt images. Zhang and Gao (2016) proposed an image encryption method which used hyper-chaotic system to control the DNA complement operation. However, because they adopted the technique of fixed DNA coding and fixed operation rules, the security of their algorithm was quite fragile. Further, the encryption key had not been associated with the original image. As a result, although complex chaotic systems was used to improve the security of the algorithm, the encrypted images could still be easily deciphered by the CPA and brute force attack (BFA) or the known plaintext attack (KPA) (Dou et al., 2017). Note that chaotic systems play a major role in such encryption methods, while the DNA coding operation without chaotic mapping was equivalent to the calculation of binary bits, and its security was not guaranteed. For example, Kumar et al. (2016) proposed a technique using DNA coding combined with elliptic curve Diffie-Hellman for image encryption, while it was deciphered by Akhavan et al. (2017) at no much cost using the chosen plaintext attack.

For these reasons, researchers used more efficient DNA coding mechanisms and DNA operations to achieve better performance chaotic systems for image encryption. In terms of DNA coding, Kalpana and Murali (2015), Zhen et al. (2016), Chai et al. (2017), Rehman et al. (2018), Dagadu et al. (2019a), and Hossein et al. (2020), etc. proposed different DNA dynamic coding, respectively. These methods gave DNA bases higher levels of encryption. However, all of the above dynamic DNA coding were based on image blocks or based on pixel-by-pixel. In addition, some of them (Dagadu et al., 2019a; Hossein et al., 2020) could not resist CPA. In terms of the DNA operations, dynamic addition operation (Zhang J. et al., 2016) and complement operation (Belazi et al., 2019), and cellular automata operation (Zhou et al., 2016; Chai et al., 2017) were proposed. Their encryption effects were better but the algorithms were more complex. In terms of the chaotic system, because the hyper-chaotic system obtained by fractional order calculation had low correlation and more complex dynamic characteristics (Zhu et al., 2014), it was favored by researchers. For example, Zhang L.M. et al. (2017) used the fractional-order hyper-chaotic system (FOHC) to scramble the DNA sequence, and achieved better image encryption effect. Li et al. (2017) used the fractional-order Lorenz hyper chaotic mapping (FOLHC) to direct the DNA operations (XOR, addition, subtraction). However, these methods were complex and the keys used were independent of the original images.

In this article, a medical image protection method based on dynamic deoxyribonucleic acid chain operation is proposed. The algorithm is tested against three kinds of medical images, and the performance, safety, efficiency of the developed algorithm evaluated against existing algorithms reported in the literature. The general arrangement for this article is as follows: First, DNA dynamic coding, FOCHC, and DNA chain operation are introduced in section “Background Knowledge.” Then, section “The Proposed Algorithm” introduces the proposed method. Next, section “Simulation Results” simulates the results. Security analyses are shown in the section “Security Analyses” and the conclusion in the section “Conclusion.”

## Background Knowledge

### DNA Coding Rule

There are four bases in a deoxyribonucleic acid chain. They are adenine (A), cytosine (C), guanine (G), thymine (T), in which A and T complement with each other, so do C and G. The four bases are denoted by the binary numbers of 00, 01, 10, and 11, normally. A total of 24 types of coding have been list (Xue et al., 2010b). However, because 0 and 1 complement with each other in binary, so do 01 and 10, and 00 and 11. Thus, only 8 of the 24 DNA coding rules satisfy the principle of base complementary, as shown in Table 1.

**TABLE 1:** Eight kinds of DNA coding rules.

**Binary**	**R1**	**R2**	**R3**	**R4**	**R5**	**R6**	**R7**	**R8**
00	A	A	C	C	G	G	T	T
01	C	G	A	T	A	T	C	G
10	G	C	T	A	T	A	G	C
11	T	T	G	G	C	C	A	A

By summarizing and categorizing the existing dynamic coding cases, it is observed that the existing cases fall into three categories: (1) those are based on the image block (column/row) dynamic coding (Akhavan et al., 2017); (2) those are based on the pixel dynamic encoding (Kalpana and Murali, 2015; Dagadu et al., 2019b; Wang X.Y. et al., 2020); and (3) those are based on the binary bit dynamic coding (Zhang J. et al., 2016). Because a DNA chain contains four bases, theoretically each base should appear in a random image with 25% of probability. The following equation can be used to calculate the base distribution rate of the above different DNA dynamic coding. The result is shown in Table 2.

**TABLE 2:** The base distribution rate of different kinds of dynamic coding.

**Base distribution**	**By row (%)**	**By pixel (%)**	**By bit (proposed) (%)**
AP	24.50	24.90	24.98
TP	24.65	24.90	25.04
CP	25.42	25.33	24.97
GP	25.43	24.87	25.01

(1) A⁢P=c⁢o⁢u⁢n⁢t⁢(A)÷(M×N×4)×100%

This equation uses base “A” as the example, where M and N are the numbers of row and column in the image; count (A) is the counting function of base “A.” A pixel consists of eight bits of binary, so four bases can represent one pixel. Here M × N × 4 is the total number of the possible base appearance. The distribution rate of “T,” “C,” and “G” can be calculated similarly.

**FIGURE 1:**
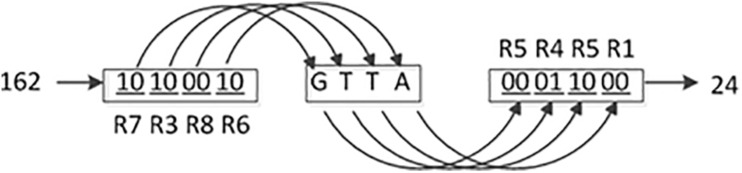
The process diagram of DNA dynamic encoding and decoding by binary bit.

From Table 2, it is found that the values of the base distribution rate of the DNA dynamic coding by binary bit are close to 25%, and the maximum deviation of the base distribution rates from 25% is 0.04%. Consequently, the DNA dynamic coding by binary bit is used for encoding and decoding in this study. A detailed coding example is shown in Figure 1. Where (R7, R3, R8, and R6) and (R5, R4, R5, and R1) are the encoding and decoding rules from Table 1, respectively, and they are controlled by the chaotic map. It can be seen that the image pixel value changes from 162 to 24. To our knowledge, this is the best DNA coding for image encryption.

### Fractional-Order Chen Hyper Chaotic (FOCHC) System

It is well known that hyper chaotic systems have much advantage over low-dimensional chaotic systems or multi-chaotic combination systems. Also the fractional-order hyper chaotic systems are superior to integer-order hyper chaotic systems in several aspects, including cross-correlation, self-correlation amplitude, pseudo-randomness, and the correlation and so on (Zhu et al., 2014).

Among the common fractional-order decomposition algorithms to solve the fractional-order chaotic system, the Adomian decomposition algorithm is the best choice since it has high precision, low complexity, and high computational efficiency (Donato and Giuseppe, 2008). Thus the Adomian decomposition algorithm is chosen to solve the FOCHC in this study, and the generated chaotic sequence is then used for image encryption. The FOCHC system model is described below:

(2) $$\left\{ \begin{array}{c} \frac{d^{q}}{d\,t^{q}}\,x=a\,\left(y-x\right)+w\\ \frac{d^{q}}{d\,t^{q}}\,y=b\,x-x\,z+c\,y\\ \frac{d^{q}}{d\,t^{q}}\,z=x\,y-d\,z\\ \frac{d^{q}}{d\,t^{q}}\,w=y\,z-e\,w \end{array}\right.$$

**FIGURE 2:**
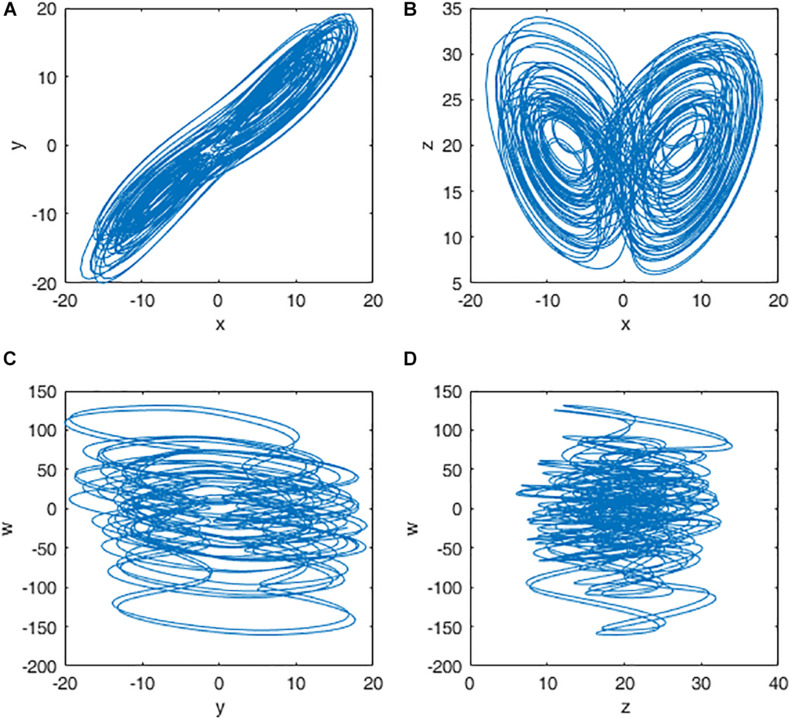
Attractors of the fractional-order Chen hyper chaotic system: **(A)** x-y plane; **(B)** x-z plane; **(C)** y-w plane; and **(D)** z-w plane.

When a=38,b=7,c=12,d=3,e=0.7, the system is in chaotic state and four chaotic sequences x,y,z,w are generated. The chaotic attractors for q = 0.98 are shown in Figure 2.

### The Definition of DNA Chain Operation

The DNA chain is defined as:

$$C_{n}=C_{h}C_{h-1}\ldots C_{2}C_{1}\left(h\leq n\right)$$

**FIGURE 3:**
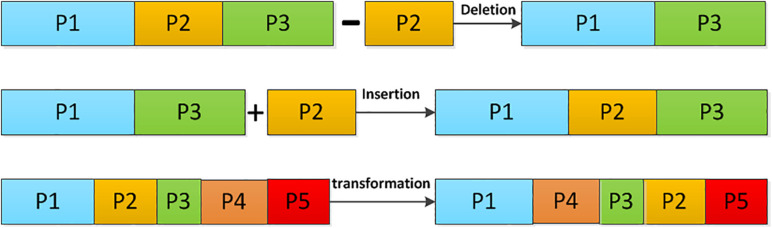
The operating principle of DNA chains.

Here, *C_n_* is a DNA chain with length m. It can be broken into smaller DNA chains of *C_h_*,*C_h_*−1,…*C*_2_,*C*_1_, with different lengths of *L*_*h*_,*L*_*h*−1_…*L*_2_,*L*_1_, respectively. Apparently, *m*=*L*_*h*_, + *L*_*h*−1_+…*L*_2_+*L*_1_. In the DNA computing, there are several operations on the DNA chain to achieve base scrambling, including the deletion, the insertion, and the transposition operations. Their operating principles are shown in Figure 3.

## The Proposed Algorithm

**FIGURE 4:**
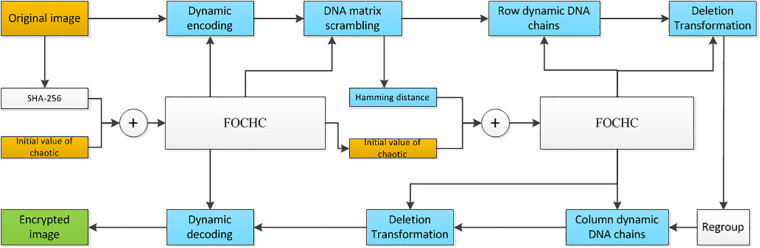
The flowchart of the proposed algorithm: yellow blocks are the input, green block is the output, and blue blocks from the DNA coding algorithm.

The proposed new method in this study includes the following four steps. Firstly, the original image is encoded into a DNA matrix dynamically, by using a FOCHC sequence. Secondly, the DNA matrix is scrambled by two other FOCHC sequences. Thirdly, DNA dynamical chain operations are carried out by four FOCHC sequences. At last, the DNA matrix generated is decoded into a binary matrix by a FOCHC sequences, and the encrypted image is obtained after recombining the DNA chain. Eight chaotic sequences are used to complete the above four steps. The eight sequences are generated by two FOCHC under different keys, which are obtained using the SHA-256 algorithm and the hamming distance. The detailed steps and the flowchart are shown in section “Key Generation,” section “Key Generation by SHA-256,” section “Key Generation by Hamming Distance, Generation of FOCHC sequences,” section “Scrambling of the DNA sequence matrix,” section “The proposed algorithm Based on the DNA dynamic chains operation,” section “Generation of the dynamic DNA chains,” section “Deletion operation on the dynamic DNA chain,” section “Transposition operation on the dynamic DNA chain,” section “Insertion operation on the dynamic DNA chain,” and section “The proposed algorithm” and Figure 4.

### Key Generation

Two kinds of keys are used as the initial values of the FOCHC sequences, and they are generated by the SHA-256 algorithm and the hamming distance, respectively. The former effectively defends against the KPA and CPA, and the latter enhances the diffusion ability of the bases.

#### Key Generation by SHA-256

The key generation in this algorithm depends on the SHA-256 function proposed in Belazi et al. (2019), because the result of the SHA-256 function is sensitive to the original image changes, since even one pixel change can result in a completely different hash value. In this study the 256-bit hash value is obtained by applying the SHA-256 function to the original image at first. The value is then converted into decimal numbers in groups of eight bits, and a decimal sequence K of length 32 is obtained, which can be expressed as K={*K1*,*K2*…*K32*}. The initial values are obtained via K; the detail equation is defined below.

(3) $$\left\{ \begin{array}{c} k\,1=\left(K\,1\,⊕K\,2\,⊕K\,3\,⊕K\,4\right)/256\\ k\,2=\left(K\,5\,⊕K\,6\,⊕K\,7\,⊕K\,8\right)/256\\ k\,3=\left(K\,9\,⊕K\,10\,⊕K\,11\,⊕K\,12\right)/256\\ k\,4=\left(K\,13\,⊕K\,14\,⊕K\,15\,⊕K\,16\right)/256\\ k\,5=\left(K\,17\,⊕K\,18\,⊕K\,19\,⊕K\,20\right)/256\\ k\,6=\left(K\,21\,⊕K\,22\,⊕K\,23\,⊕K\,24\right)/256\\ k\,7=\left(K\,25\,⊕K\,26\,⊕K\,27\,⊕K\,28\right)/256\\ k\,8=\left(K\,29\,⊕K\,30\,⊕K\,31\,⊕K\,32\right)/256 \end{array}\right.$$

(4) $$\left\{ \begin{array}{c} x\,\_\,s=k\,1+x\,0\\ y\,\_\,s=k\,2+y\,0\\ z\,\_\,s=k\,3+z\,0\\ w\,\_\,s=k\,4+w\,0 \end{array}\right.$$

Where x_s,y_s,z_s,w_s are the initial values obtained, and x0, y0, z0, and w0 are the initial values given.

#### Key Generation by Hamming Distance

There are four steps for key generation by the hamming distance, as detailed in the following:

Step 1: For a DNA matrix A_DNA_maxtrix(m,n×4), calculate the hamming distance for every two rows and every two columns of the matrix, respectively. The row hamming distance R_H_={r*_h1_*,r*_h2_*…,r_hi_,…r_*hm*/2_} and the column hamming distance C_H_={c*_h1_*,c*_h2_*…,c_hi_…c_hn=4/2_} are obtained. The equation for calculating the hamming distance is:

(5) {D⁢(M,N)=∑i=0Ld⁢(m′i,⁢n′i)d⁢(m′i,⁢n′i)={0,i⁢f⁢m′i=n′i1,i⁢f⁢m′i≠n′i

Where $m_{\text{i}}^{\prime }$ and n′i are the ith base of the DNA chains M and N, respectively, and D(M,N) is the hamming distance between M and N;
Step 2: Calculate the average values of R_H_ andC_H_, which are ${\text{R}}_{\text{h}}^{\prime }$ and ${\text{C}}_{\text{h}}^{\prime }$, respectively.
Step 3: Extract the decimal parts p and q from ${\text{R}}_{\text{h}}^{\prime }$ and ${\text{C}}_{\text{h}}^{\prime }$.
Step 4: The new initial values of FOCHC are obtained by Eq. (6). x0′,y0′,z0′, and w0′ are the given initial values, *k5*,*k6*,*k7*,*k8* are calculated as described in section “Key Generation by SHA-256.”

(6) $$\left\{ \begin{array}{c} X_{h}=k\,5+p+x\,0^{\prime }\\ Y_{h}=k\,6+p+y\,0^{\prime }\\ Z_{h}=k\,7+q+z\,0^{\prime }\\ W_{h}=k\,8+q+w\,0^{\prime } \end{array}\right.$$

### Generation of FOCHC Sequences

The initial values generated using the FOCHC sequences, as described in section “Key generation,” was input into the FOCHC system to produce four groups of chaotic sequences X,Y,Z,W after being iterated for 1000+m×n×4 times. To eliminate the transient effects in the chaotic systems, the chaotic sequences were recalculated for 1,000 iterations before being used, and their length were m×n×4.

### Scrambling of the DNA Sequence Matrix

Step 1: Input the DNA sequence matrix A (m, n ×4), whose size is (m,n ×4);
Step 2: Use the following equations to transform the chaotic sequences:

(7) $$\left\{ \begin{array}{c} Y\,Y=a\,b\,s\,\left(Y\,1-f\,i\,x\,\left(Y\,1\right)\right)\\ Z\,Z=a\,b\,s\,\left(Z\,1-f\,i\,x\,\left(Z\,1\right)\right) \end{array}\right.$$

Where, Y1 and Z1 are the FOCHC sequences. The length of Y1 is m, and the length of Z1 is n ×4.fix(.) is the rounding function. abs(.) is the absolute value function.
Step 3: Sort YY and ZZ, respectively, to obtain the index values By and Bz.

(8) $$\left\{ \begin{array}{c} \left[∼,B\,y\right]=s\,o\,r\,t\,\left(Y\,Y\right)\\ \left[∼,B\,z\right]=s\,o\,r\,t\,\left(Z\,Z\right) \end{array}\right.$$

Step 4: Use the following equation to scramble A, and obtain the matrix A_scrambing.

(9) A⁢_⁢s⁢c⁢r⁢a⁢m⁢b⁢i⁢n⁢g⁢(i,j)=A⁢(B⁢y⁢(i),B⁢z⁢(j));

Where i=1,2…m,j=1,2…n×4.

### The Proposed Algorithm Based on the DNA Dynamic Chains Operation

**FIGURE 5:**
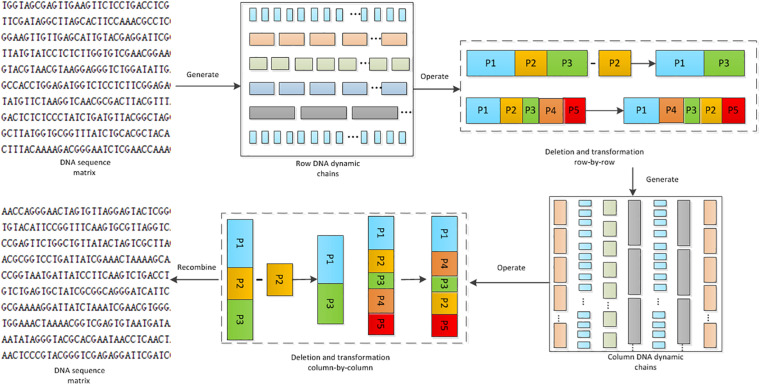
Flowchart of the DNA dynamic chain operations.

Through DNA dynamic chain operation, the algorithm proposed changes the position of the base, which leads to changes of the pixel values in the image. As shown in Figure 5, each row in the DNA sequence matrix is divided into chains of different lengths. Deletion operation and transposition operation are applied on these chains. This is the first shuffle process on the DNA sequence matrices. To eliminate the block effect, generation of the column DNA dynamic chain, and the deletion and transposition operations are conducted again. Thus the DNA sequence matrices are scrambled for the second time. In the whole process, the length of the DNA chain is dynamic, so is the operation, and the DNA base is completely disrupted.

#### Generation of the Dynamic DNA Chains

DNA sequence matrix is divided into DNA chains of different lengths by rows or columns. The length of the chain is controlled by the hyper-chaotic sequence. The following explains the detailed steps for generating DNA dynamic chains row-by-row. Generation of the DNA dynamic chains column-by-column can be implemented in a similar way, except that the DNA matrix needs to be transposed before the generation.

Step 1: A FOCHC sequence x1 is transformed using the following equation:

(10) $$x\,1=m\,o\,d\,\left(f\,i\,x\,\left(a\,b\,s\,\left(x\,1-f\,i\,x\,\left(x\,1\right)\right)\times 10^{14}\right),4\right)+1$$

Where, fix(.) is the rounding function, and abs(.) is the absolute value function.
Step 2: The length of chains a(i) is determined with the following equation:

(11) $$\text{a}\,\left(\text{i}\right)=\left\{ \begin{array}{c} 16,\mathrm{if}\,\mathrm{x1}\,\left(\text{i}\right)=1\\ 8,\mathrm{if}\,\mathrm{x1}\,\left(\text{i}\right)=2\\ 4,\mathrm{if}\,\mathrm{x1}\,\left(\text{i}\right)=3\\ 2,\mathrm{if}\,\mathrm{x1}\,\left(\text{i}\right)=4 \end{array}\right.$$

Where, i=1,2,3…m,m is the size of the rows in the DNA sequence matrix.
Step 3: DNA dynamic chain matrices are obtained by decomposing each row of the DNA matrix sequences, according to different lengths, as shown in the following equation:

(12) R⁢o⁢w⁢_⁢c⁢h⁢a⁢i⁢n=D⁢N⁢A⁢_⁢d⁢e⁢c⁢o⁢m⁢p⁢o⁢s⁢e⁢(D⁢N⁢A⁢_⁢m⁢a⁢t⁢r⁢i⁢x⁢(i,:),a⁢(i))

Where DNA_decompose(.) is a generation function of the DNA dynamic chain, which means that the ith row in the DNA_matrix is decomposed into chains whose lengths are defined in a(i).

##### Deletion Operation on the Dynamic DNA Chain

Deletion operation on the DNA dynamic chains for each row or column is implemented using the chaotic sequence. The deletion operation function *deletion*(A,X) is defined as following:
In the function *deletion*(A,X), A is a chain set of a row, which can be represented asA={a_1_,a_2_,a_3_…a_n_}, where n is the number of chains. a_i_ is the ith DNA chain. X is a chaotic sequence, which can be represented as X={x_1_,x_2_,x_3_…x_n_}. x_i_(x_i_ ∈ (0,1)) is the ith element in the chaotic sequence. Note that the length of the chaotic sequence and that of the DNA chain set are the same. Carry out deletion for a_i_ when x_i_ < 0.5, otherwise save the chain. Supposing that the a_i_ chain has been deleted, the a_i_ chain is moved to the end of the DNA chain. Other deleted chains can be processed in the same way.

#### Transposition Operation on the Dynamic DNA Chain

Transposition operation on the DNA dynamic chains for each row or column is conducted using the chaotic sequence. The Transposition operation function *Transposition*(A,X) is defined as following:

In the function *Transposition*(A,X), the definitions of A and X are the same as those in section “Deletion Operation on the Dynamic DNA Chain.” A new sequence X′ is obtained by transposing X, with a_i_ and ${\text{a}}_{{\text{i}}^{\prime }}$ exchanged, where i′ is the location of the ith element in X′.

#### Insertion Operation on the Dynamic DNA Chain

Insertion operation is used for the decryption process, the insertion operation function insertion (A,X) is defined as following:

In the function insertion (A,X), A is a chain set of a row, which can be represented as A={a_1_,a_2_,a_3_…a_n_} where n is the number of chains. a_i_ is the ith DNA chain. X is a chaotic sequence, which can be represented as X={x_1_,x_2_,x_3_…x_n_}. x_i_(x_i_ ∈ (0,1)) is the ith element in the chaotic sequence. Note that the length of the chaotic sequence and that of the DNA chain set are the same. Set*count* = 0, when x_i_ < 0.5, carry out count=count + 1, count is the number of deleted DNA chains in the encryption process. Carry out *e*_*j*_=*a*_*n*−*count* + *j*_, where *j* = 1,2,…*count*, here *e_j* is the *jth* deleted DNA chain. Set *q* = 1, j = 1, if x_i_ < 0.5, *f*_*i*_=*e*_*j*_,*j* + + ; else *f*_*i*_=*a*_*q*_,*q* + +. A1 = {f_1_,f_2_,f_3_…f_n_} is obtained after insertion operation.Other inserted chains can be processed in the same way.

### The Proposed Algorithm

The detailed steps of the proposed algorithm are listed below.

Step 1: Input the initial values x0,y0,z0,w0 and an 8-bit image A (m, n), where m and n define the size of the image. A binary matrix A′(m, n × 8) is obtained by transforming A (m, n).
Step 2: Use the SHA-256 function to generate the chaotic initial values x_s,y_s,z_s,w_s, as explained in section “Key Generation.”
Step 3: Produce four chaotic sequences X, Y, Z, W using FOCHC with the initial values x_s,y_s,z_s,w_s, as detailed in section “Generation of FOCHC Sequences.”
Step 4: Generate the matrix A_encode(m,n×4) using the chaotic sequence X1 to encode A′(m, n × 8), as detailed in section “DNA Coding Rule.” X1 is obtained using the following equation:

(13) $$X\,1=m\,o\,d\,\left(f\,i\,x\,\left(a\,b\,s\,\left(X-f\,i\,x\,\left(X\right)\right)\times 10^{14}\right),8\right)+1$$

Where fix(.) is the rounding function, and abs(.) is the absolute value function.
Step 5: Scramble A_encode(m,n×4) by using two chaotic sequences Y,Z, as explained in section “Scrambling of the DNA Sequence Matrix.” This produces the matrix A_DNA_scrambling(m,n×4).
Step 6: Calculate the hamming distance of A_DNA_scrambling to obtain the new initial values x_h,y_h,z_h, w_h, as explained in section “Key Generation by Hamming Distance,” which are then used to generate four chaotic sequences X′,Y′,Z′,W′.
Step 7: Divide A_DNA_scrambling into different lengths of DNA dynamic chains row-by-row by using the chaotic sequence X′, as explained in section “Generation of the Dynamic DNA Chains.” This produces the DNA chain matrix A_Row_Chain.
Step 8: Conduct the deletion and transposition operations on A_Row_Chain using the chaotic sequence Y′, as described in section “Deletion Operation on the Dynamic DNA Chain” and section “Transposition Operation on the Dynamic DNA Chain.” After recombining the data, this produces the matrix A_Row_operation.
Step 9: Divide A_Row_operation into different lengths of DNA dynamic chains column-by-column using the chaotic sequence Z′, as described in section “Generation of the Dynamic DNA Chains.” This produces the DNA chain matrix A_Column_Chain.
Step 10: Conduct the deletion and transposition operations on A_Column_Chain using the chaotic sequence W′, as explained in section “Deletion Operation on the Dynamic DNA Chain” and section “Transposition Operation on the Dynamic DNA Chain.” After recombining the data, this produces the matrix A_Column_operation.
Step 11: Decode the matrix A_Column_Chain dynamically using the chaotic sequence*W1*, as explained in section “DNA Coding Rule.” This produces the new matrixA_decode. *W1* is calculated using the following equation:

(14) $$W\,1=m\,o\,d\,\left(f\,i\,x\,\left(a\,b\,s\,\left(W-f\,i\,x\,\left(W\right)\right)\times 10^{14}\right),8\right)+1$$

Step 12: RecombineA_decode to obtain the encrypted image B.
The decryption algorithm is the inverse of the encryption algorithm detailed above; also the delete operation needs to be replaced with the insert operation.

## Simulation Results

The proposed algorithm explained above is then tested on three kinds of medical images of MRI, CT, and X-ray. All of the experimental data are 512 × 512 images extracted from the database^1^. Matlab 2019a is used to code the proposed algorithm, and the code is running in the 64-bit Window 7 environment with 8GB RAM and the i5-7200U CPU. The keys of the encryption algorithm presented in this article are composed of the hash value, the row and column hamming distance values, and two sets of chaotic initial values, as shown in Table 3. Table 4 lists the experimental results using the extracted images.

**TABLE 3:** The key of the proposed algorithm.

**Composition of the key**	**The key of encryption and decryption**
Hash value	c515f75a2b612d728e3356b7b53925 32044172d647291f10f00075107f161bd9
Hamming distance	Rh′=393425,Ch′=393058
Initial value of two FOCHC system	x0 = 0.12,y0 = 0.35,z0 = 0.68,w0 = 0.42, x0′ = 0.37,y0′ = 0.54,z0′ = 0.89,w0′ = 0.76

**TABLE 4:** Results of the proposed algorithm.

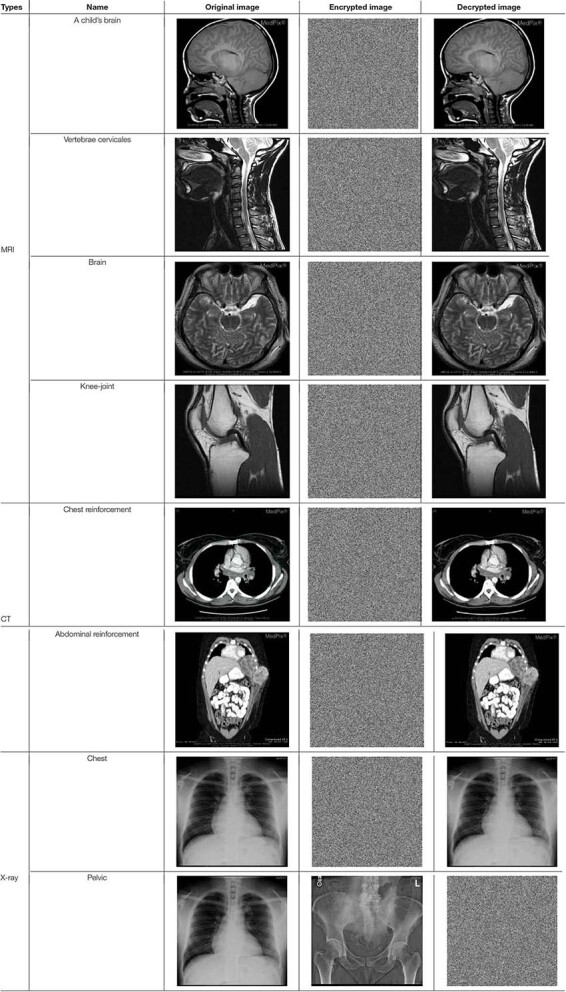

As illustrated in Table 4, no any useful information can be drawn from the encrypted images for all the three types of medical images tested, while the decrypted images show no difference when compared with the original images. From the point of view of visual inspection, the proposed algorithm worked satisfactorily.

### Security Analyses

#### Key Space Evaluation

Section “Simulation Results” shows that the keys consist of three parts: the hash value, the hamming distances and the chaotic initial value. Therefore, in addition to the hash value and the hamming distances, there are another eight keys in the proposed algorithm. They are x0, y0, z0, w0, x0′, y0′, z0′, and w0.′ All of them have 14 bits precision, so the key space is (10^14^)^7^=10^112^≈2^372^. The SHA-256 value with the complexity of the finest attack is 2^128^. All of them are larger than 2^100^ (Wang X.Y. et al., 2020). Thus the key space is large enough to withstand BFA. Moreover, generation of the key depends on the original image, which forms a one-time pad shame, and makes it difficult for the attacker to predict the encryption key.

#### Key Sensitivity Evaluation

**FIGURE 6:**
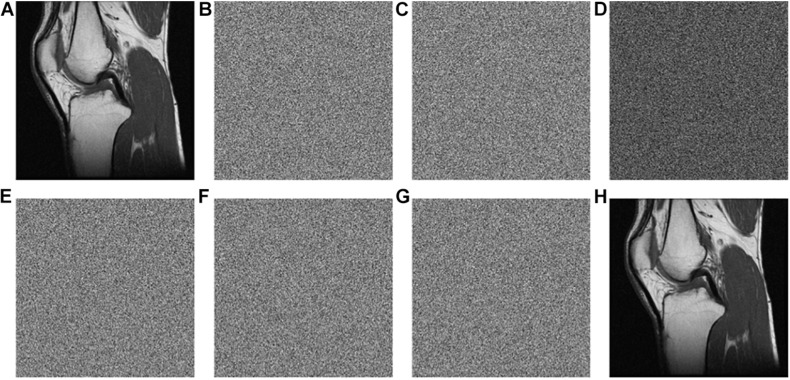
Test of key sensitivity. **(A)** The original image of “MRI-knee joint.” **(B)** The encrypted image with the initial key set. **(C)** The encrypted image with x0+t. **(D)** The differential image between **(B,C)**. **(E)** The decrypted image with x0+t. **(F)** The decrypted image with ${\text{R}}_{\text{h}}^{\prime }+1$. **(G)** The decrypted image with the hash value + “1.” **(H)** The decrypted image with the correct key.

The keys are used in the encryption and decryption process, and the key sensitivity means that when the encryption keys each change slightly, the image generated will be completely different to the initial encrypted image. Similarly, when the decryption keys each change slightly, the correct decryption image cannot be obtained. This article tests the sensitivity of the keys from the aspects of encryption and decryption separately. Figure 6 and Table 5 show the results.

**TABLE 5:** Difference rate of two encrypted image obtained by slightly different keys.

**Image**	**Difference rate (%)**								
	**x0+t**	**y0+t**	**z0+t**	**w0+t**	**x0′+t**	**y0′+t**	**z0′+t**	**w0′+t**	**SHA-256+1**
MRI-knee	99.6101	99.6326	99.6025	99.6014	99.5232	99.5304	99.5224	99.5136	99.6132
CT-abdominal	99.5964	99.6101	99.6307	99.6044	99.5209	99.4858	99.5049	99.5335	99.6120
X-ray-Pelvic	99.6227	99.6174	99.6014	99.6106	99.5197	99.5182	99.5537	99.5308	99.6212
Average	99.6097	99.6200	99.6115	99.6054	99.5220	99.5115	99.5270	99.5260	99.6155

Figure 6B is the encrypted image of the “MRI-Knee-joint” with the keys listed in Table 3. Figure 6C is the same as Figure 6B beside x0 is changed to x0+t, where *t* = 0.00000000000001. Figure 6D shows that Figures 6B,C have much difference. To further observe the sensitivity of the keys, one key is changed slightly (add t) and the other keys kept unchanged, after that, these keys are used to encrypt the images “MRI-knee,” “CT-abdominal,” and “X-ray-pelvic,” respectively. At last, the difference rate of the encrypted images before and after the slight key changes is calculated. All the average difference rates in Table 5 are above 99.50%, which is very close to 100%. On the other hand, the original images are restored using three types of keys (x0,R_h_’ and the hash value) changed slightly. Figure 6E is the decrypted image with x0 = x0+t and other keys unchanged. Figure 6F is the decrypted image with x0 = ${\text{R}}_{\text{h}}^{\prime }+1$ and other keys unchanged. Figure 6G is the decrypted image with the hash value + ‘1’ and other keys unchanged. Figures 6E–G show that the original image cannot be restored when the keys change slightly. The original image can only be recovered when the keys are correct, as shown in Figure 6H. This test proved that the key sensitivity is very high, and the algorithm is robust against the exhaustive attacks.

### Statistical Analysis Evaluation

#### The Histogram Evaluation of the Decrypted Image

The distribution of the histogram is evaluated by observing the histogram for the encrypted image and calculating the variance of the histogram. The more uniform the histogram distribution for the encrypted image, the stronger the ability of anti-statistical analysis. The variance of the histogram is defined as follow:

(15) $$V\,a\,r\,\left(M\right)=\frac{1}{n^{2}}\times \sum_{i=1}^{n}\sum_{j=1}^{n}\frac{1}{2}\,{\left(m_{i}-m_{j}\right)}^{2}$$

Where n is the gray scale value, here it is set as *n* = 256. m_i_ is the number of pixels whose gray values are equal to i, with i being the value in the histogram value. m_i_ is the same as m_j_.

**FIGURE 7:**
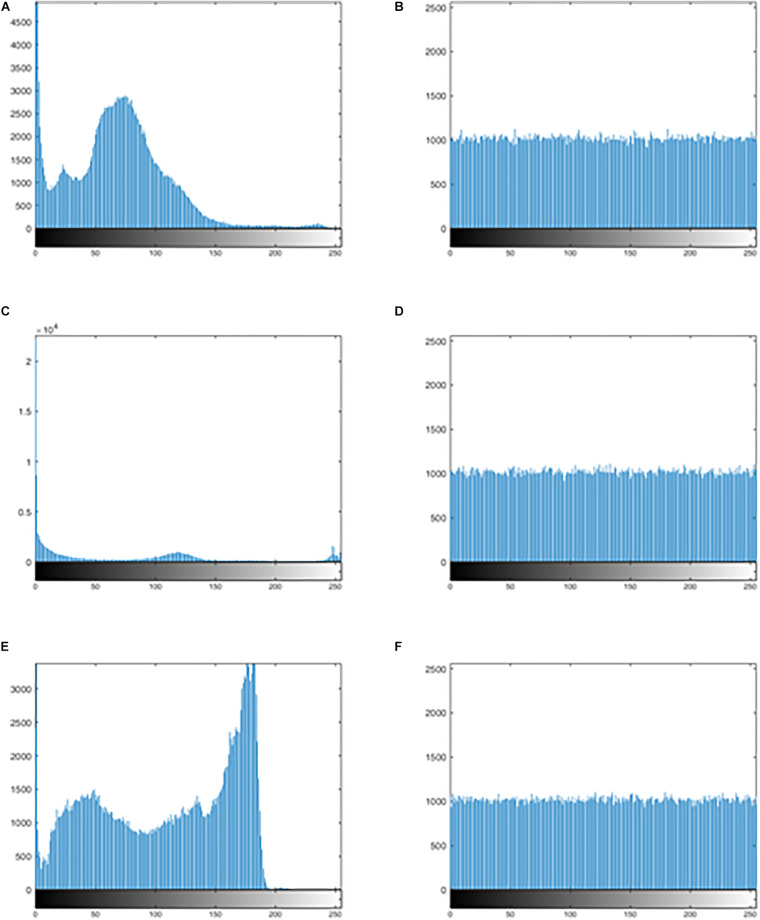
The histogram of the original image and the encrypted image. **(A)** The histogram of the original image for “MRI-brain.” **(B)** The histogram of the encrypted image for the “MRI-brain.” **(C)** The histogram of the original image for the “CT-chest.” **(D)** The histogram of the encrypted image for the “CT-chest.” **(E)** The histogram of the original image for the “X-ray-chest.” **(F)** The histogram of the encrypted image for the “X-ray-chest.”

Figures 7A,C,E show the histogram of three kinds of original images, and Figures 7B,D,F show the histogram corresponding to the images after encrypting. Observation suggests that Figures 7B,D,F looks very uniform. In Table 6, the variance values for the encrypted images are significantly reduced. In addition, the average of the variance values is lower than other algorithm, as detail in the Table 7. In summary, it is difficult to extract original information through statistical analysis on the histogram.

**TABLE 6:** The variance of the histogram.

**Image**	**Original image**	**Encrypted image**
MRI-child’s brain	1.9859 × 10^7^	1.0956 ×10^3^
MRI-cervical vertebra	2.0992 × 10^6^	1.0379 ×10^3^
MRI-brain	2.8428 × 10^6^	1.1222 ×10^3^
MRI-knee-joint	2.6840 × 10^6^	871.1016
CT-chest	8.0452 × 10^7^	1.0736 ×10^3^
CT-abdominal	4.4843 × 10^7^	907.8516
X-ray-chest	7.8361 × 10^5^	1.0098 ×10^3^
X-ray-pelvic	2.0195 × 10^6^	843.0469
Average	2.1938 × 10^7^	995.1375

**TABLE 7:** The variance of the histogram comparison.

**Algorithm**	**Variance**
**Proposed**	**995.1375**
Chai et al., 2019	1051
Liu et al., 2019	1341

#### Correlation Coefficient Evaluation

Usually, the recognizable images have high correlation, so correlation evaluation is one of the effective means to measure the encryption effect. The closer the correlation coefficient to 0, the better the encryption result. The correlation coefficient is defined as follows:

(16) $$r_{x\,y}=\frac{\begin{array}{c} \frac{1}{N}\times \sum_{i=1}^{N}\left(x_{i}-\frac{1}{N}\times \sum_{i=1}^{N}x_{i}\right)\\ \left(y_{i}-\frac{1}{N}\times \sum_{i=1}^{N}y_{i}\right) \end{array}}{\begin{array}{c} \sqrt{\frac{1}{N}\times \sum_{i=1}^{N}\left(x_{i}-{\left(\frac{1}{N}\times \sum_{i=1}^{N}x_{i}\right)}^{2}\right)}\\ \sqrt{\frac{1}{N}\times \sum_{i=1}^{N}\left(y_{i}-{\left(\frac{1}{N}\times \sum_{i=1}^{N}y_{i}\right)}^{2}\right)} \end{array}}$$

Where x_i_ and y_i_ are adjacent pixels selected randomly in three directions (horizontal, vertical, and diagonal). For evaluation 8,000 pairs of adjacent pixels are chosen for the test.

**FIGURE 8:**
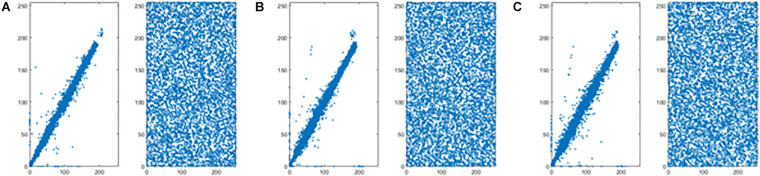
Correlation of adjacent pixels of the original image and the encrypted image for “CT-chest.” **(A)** Horizontal direction. **(B)** Vertical direction. **(C)** Diagonal direction.

Figures 8A–C shows the correlation coefficients in the three directions, respectively. Obviously, the distribution of point sets is concentrated in the left subfigure of Figures 8A–C. On the contrary, the distribution of point sets is discrete in the right subfigure of Figures 8A–C. The values of correlation coefficients are shown in Table 8. The correlation coefficients of the encrypted image are very close to 0. From the comparison results, it is also better than other algorithms, which are shown in Table 9. This sufficiently demonstrates that it is difficult for attackers to obtain a cipher image by statistical pixel correlation.

**TABLE 8:** Correlation coefficients of two adjacent pixels of the original and the encrypted images.

**Image**	**Original image**			**Encrypted image**		
	**H**	**V**	**D**	**H**	**V**	**D**
MRI-child’s brain	0.9757	0.9806	0.9563	0.0013	6.5787 ×10^−4^	−0.0049
MRI-cervical vertebra	0.9714	0.9738	0.9495	−1.6397 ×10^−4^	9.0951 ×10^−4^	−0.0015
MRI-brain	0.9812	0.9809	0.9607	0.0036	−0.0075	0.0021
MRI-knee-joint	0.9928	0.9970	0.9912	−9.6693 ×10^−4^	−6.6741 ×10^−4^	4.7456 ×10^−4^
CT-chest	0.9746	0.9603	0.9455	0.0012	−2.7242 ×10^−4^	0.0011
CT-abdominal	0.9788	0.9827	0.9656	−9.1875 ×10^−4^	3.8182 ×10^−4^	−0.0018
X-ray-chest	0.9946	0.9779	0.9774	0.0011	6.4868 ×10^−4^	0.0049
X-ray-pelvic	0.9340	0.9529	0.9114	0.0012	−2.0257 ×10^−4^	−4.7550 ×10^−4^

**TABLE 9:** Correlation coefficients comparison.

**Algorithm**	**H**	**V**	**D**

**Proposed (CT-chest)**	**0.0012**	−**0.0003**	**0.0011**
Hossein et al., 2018 (medical image)	0.0031	0.0029	0.0013
Belazi et al., 2019 (medical image)	0.0013	−0.0049	0.0057
Dagadu et al., 2019a (medical image)	−0.0016	0.0043	−0.0061
Wang X.Y. et al., 2020	−0.0021	0.0009	0.0003
Hossein et al., 2020	0.0059	0.0029	0.0018
Wu et al., 2019	0.0158	0.0023	−0.0336
Zhang X.C. et al., 2017	0.0082	0.0032	0.0150

### Global and Local Information Entropy Evaluation

There is redundancy in any image, which is related to the probability or uncertainty of each pixel in the image. Usually, this uncertainty is measured with the global and the local information entropy (Zhang et al., 2012; Wu et al., 2013). The global information entropy is a measure of the distribution of all pixels in an image, while the local information entropy is a measure of the distribution of pixel values in an image block. Compared with the global information entropy, the local information entropy is more efficient, accurate and consistent in judging the pixel values distribution situation of the image.

It is known that the global information entropy of an ideal random image is 8. Also Wu et al., 2013 shows that the local entropy values for the ideal random image blocks of 16 × 16 and 32×32 are 7.1749 and 7.8087, respectively. Table 10 lists the global and local information entropy of all the encrypted images processed using the proposed algorithm. It is observed that the average global information entropy of all the encrypted image is 7.9993, and the average local entropy are 7.1715 for the 16 × 16 block and 7.8016 for the 32 × 32 block. All of them are close to the ideal values. Furthermore, comparison with other algorithms is shown in Table 11, which also includes the comparison of global information entropy corresponding to the image encryption algorithm. Apparently, the information entropy of the proposed algorithm in this study is superior to those for other algorithms.

**TABLE 10:** The entropy of eight medical encrypted images.

**Image**	**Entropy**	**Local entropy (16 ×16)**	**Local entropy (32 × 32)**
MRI-child’s brain	7.9992	7.1530	7.7962
MRI-Vertebrae cervicales	7.9993	7.1705	7.7995
MRI-Brain	7.9992	7.1800	7.8037
MRI-Kneejoint	7.9994	7.1935	7.8010
CT-Chest	7.9993	7.1688	7.8034
CT-Abdominal	7.9994	7.1567	7.8007
X-ray-Chest	7.9993	7.1615	7.8037
X-ray-Pelvic	7.9994	7.1883	7.8046
Average	7.9993	7.1715	7.8016

**TABLE 11:** Global information entropy comparison.

**Algorithm**	**Entropy**
**Proposed**	**7.9993**
Hossein et al., 2018 (medical image)	7.9990
Belazi et al., 2019 (medical image)	7.9974
Dagadu et al., 2019a (medical image)	7.9993
Hua et al., 2018 (medical image)	7.9981
Wang X.Y. et al., 2020	7.9971
Azimi and Ahadpour, 2020	7.9988
Hossein et al., 2020	7.9989
Wu et al., 2019	7.99895
Yang et al., 2019	7.9964

### Plaintext Sensitivity (Differential Attack)

Differential attack is one of the common attack methods used by cryptanalysts. Its main idea is to encrypt two original images with tiny change and no change, respectively, then compare the relationship between the encrypted image before and after change, and predict the encryption key, so as to decipher the encryption algorithm. NPCR and UACI in the Reference (Wang X.Y. et al., 2020) are used here to test the ability of the algorithms to resist differential attack.

The NPCR and UACI values of the encrypted images obtained from the two slightly changed images are shown in Table 12. The average values obtained are 99.6191% and 33.4815%, which are higher than the values for other algorithms, as detailed in Table 13.

**TABLE 12:** The result of differential attack (NPCR, UACI).

**Image**	**NPCR (%)**	**UACI (%)**
MRI-child’s brain	99.6124	33.4986
MRI-Vertebrae cervicales	99.6162	33.4415
MRI-Brain	99.6185	33.4285
MRI-Knee joint	99.6220	33.4547
CT-Chest	99.6254	33.4474
CT-Abdominal	99.6334	33.5747
X-ray-Chest	99.6059	33.4359
X-ray-Pelvic	99.6193	33.5704
Average	99.6191	33.4815

**TABLE 13:** Comparison of the average differential attack (NPCR,UACI) by different encryption algorithms.

**Algorithm**	**NPCR (%)**	**UACI (%)**

**Proposed**	**99.6191**	**33.4815**
Hossein et al., 2018 (medical image)	99.1349	33.1633
Belazi et al., 2019 (medical image)	99.6536	33.4121
Dagadu et al., 2019a (medical image)	99.6100	33.5075
Wang X.Y. et al., 2020	99.5956	33.4588
Wang et al., 2018	99.5700	32.3800
Hossein et al., 2020	99.5438	33.4742
Wu et al., 2019	99.5666	33.3966
Yang et al., 2019	99.6105	33.4694

### Noise Attack

**FIGURE 9:**
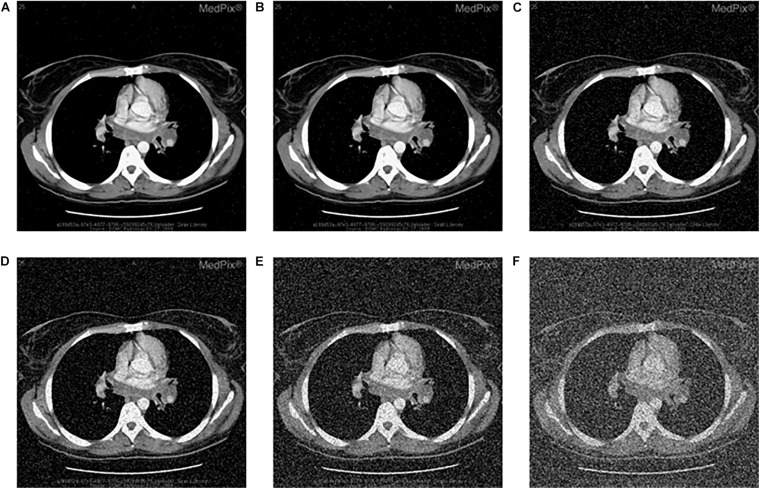
Salt-and-pepper noise attack of encrypted CT-chest, decrypted image with density value **(A)** 0.002, **(B)** 0.005, **(C)** 0.05, **(D)** 0.1, **(E)** 0.25, and **(F)** 0.5.

Images can be contaminated by noise during transmission. To analyze the anti-noise capability, the same encrypted image is attacked by the salt and the pepper noise with the density of 0.002, 0.005, 0.05, 0.1, 0.25, and 0.5, respectively, Figures 9A–F lists the decrypted images after being attacked with the salt and the pepper noise. All of them can clearly show the outline and texture of the original image. Further, equation (17) is used to calculate the PSNR between the original image and Figures 9A–F. The PSNR of the proposed algorithm is then compared with that of other algorithms. Results in Figure 9 and Table 14 show that the proposed algorithm is immune to the salt and the pepper noise.

**TABLE 14:** PSNR (db) between the original and the decrypted images under noise.

**Algorithm**	**Density of salt & pepper noise**					
	**0.002**	**0.005**	**0.05**	**0.1**	**0.25**	**0.5**

**Proposed**	**33.8870**	**29.6681**	**19.7353**	**16.6353**	**12.4552**	**9.1951**
Belazi et al., 2019	32.8396	28.7068	18.8395	15.8599	12.2262	9.8903
Zhou et al., 2015	26.1682	21.9976	12.8812	10.6900	8.8973	8.5504
Hua and Zhou, 2017	8.5900	8.5625	8.5514	8.5476	8.5454	8.5428
Hua et al., 2018	29.8380	25.6571	15.8923	13.1335	10.2166	8.8271
Liu et al., 2016	/	19.1553	19.5829	11.9524	/	/

(17) $$P\,S\,N\,R=10\,\text{lg}\,\frac{255\times 255\,\text{MN}}{\sum_{i=1}^{M}\sum_{j=1}^{N}{\left|x^{\prime }\,\left(i,j\right)-x\,\left(i,j\right)\right|}^{2}}$$

### Occlusion Attack

**FIGURE 10:**
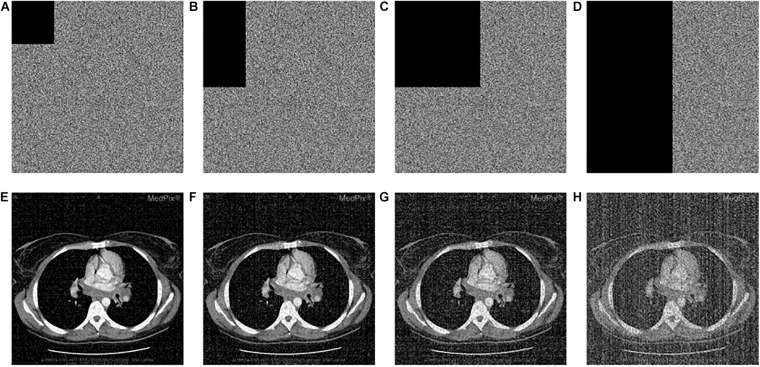
Occlusion attack: the data loss of encrypted “CT-chest” with **(A)** 1/16, **(B)** 1/8, **(C)** 1/4, and **(D)** 1/2; corresponding decrypted image **(E–H)** in accordance with **(A–D)**.

To analyze the anti-occlusion capability of the proposed algorithm, the same cipher image is occluded with 1/16, 1/8, 1/4, and 1/2, respectively. Then, the blocked images are decrypted with the proposed algorithm. Take the image “CT-chest” as the example, which are shown in Figures 10A–H. As shown, all the encrypted images which are occluded with different area are recovered successfully. In all of them the information of the original image can be identified. Additionally, comparison of the ability to resist occlusion attack for the proposed and other algorithm by PSNR is shown in Table 15. Obviously the proposed algorithm is superior to others.

**TABLE 15:** PSNR (db) between the original and decrypted images under occlusion.

Algorithm	Occlusion			
	**1/16**	**1/8**	**1/4**	**1/2**
**proposed**	**18.7275**	**15.6445**	**12.3947**	**9.1317**
Belazi et al., 2019	26.6301	17.6447	14.6193	11.6147
Zhou et al., 2015	12.0881	10.0969	8.8968	8.5539
Hua and Zhou, 2017	8.5675	8.5540	8.5502	8.5480
Hua et al., 2018	17.7218	14.8610	12.1275	9.7698

### Known-Plaintext and Chosen-Plaintext Attacks

Kerckhoffs’ principles in cryptography state that encryption and decryption algorithms are known or transparent in a cryptosystem. Therefore, the security of the cryptosystem depends on the key rather than the encryption algorithm itself. By exploring the relationship between the key and the ciphertext or the plaintext and ciphertext, the attacker obtains the valid equivalent key, and then decrypts the original image. The main methods include the ciphertext-only attack, KPA, CPA, and chosen ciphertext attack (CCA). Among these attack methods, CPA is recognized as the strongest attack method, so the ability of the current algorithm to resist CPA is analyzed here.

**FIGURE 11:**
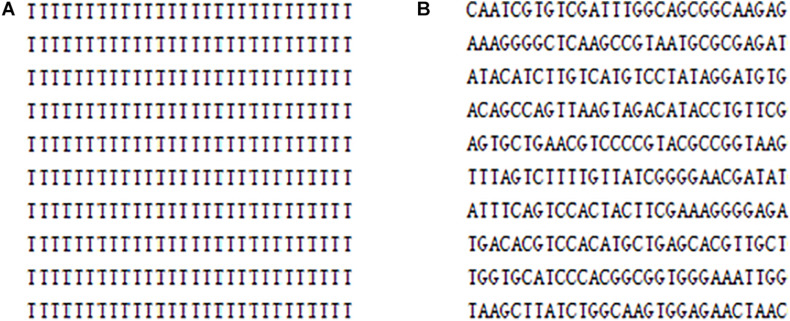
The part of DNA encoding matrix of “pure white” image: **(A)** DNA fixed encoding **(B)** DNA dynamic encoding of the proposed algorithm.

**FIGURE 12:**
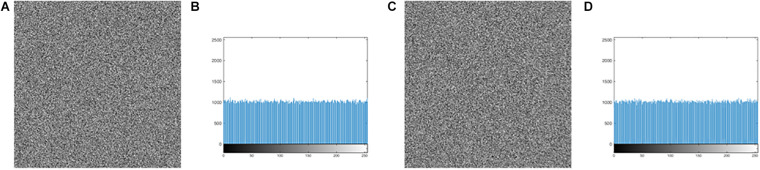
The encrypted image and histogram of “pure white” image and “pure black” image: **(A)** The encrypted image of “pure white.” **(B)** The histogram of **(A)**. **(C)** The encrypted image of “pure black.” **(D)** The histogram of **(C)**.

As for the key generation, the encryption keys of this algorithm are generated by the SHA-256 function and the hamming distances. Because the calculation of the SHA-256 function and the hamming distances are closely related to the plaintext, the key is very sensitive to the plaintext. In other words, a small change in the plaintext image produces a completely different key, as detailed in section “Key Sensitivity Evaluation.” In the DNA encoding, this article uses the DNA dynamic encoding by binary bit, compared with the traditional fixed DNA coding and other existing DNA dynamic encoding methods, the base distribution is more uniform, which can be found in Table 2. Additionally, for pure white or pure black images, encoding with DNA fixed can cause multiple base repeats, as shown in Figure 11. Clearly, Figure 11A gives an attacker an opportunity, but the DNA bases in Figure 11B are irregular. From this whole encryption system, both “pure white” and “pure black” images of the encrypted images and the corresponding histograms are derived, which are shown in Figure 12. Moreover, Figures 12A,C are evaluated in Table 16. The histogram in Figure 12 is evenly distributed. The information entropy in Table 16 is 7.9994. Their NPCR and UACI are both higher than 99.6% and 33.4%. The correlation coefficients are close to 0. It can be shown that it is difficult for an attacker to analyze the equivalent key by choosing pure white or black images. To sum up, the proposed algorithm is robust in defending against the chosen plaintext attack.

**TABLE 16:** The performance of the encrypted “pure white” image and the encrypted “pure black” image.

Encrypted image	Entropy	variance	Horizontal	Vertical	Diagonal	NPCR	UACI
White	7.9994	816.5000	0.0018	−0.0014	0.0016	99.6078	33.4375
Black	7.9994	880.3828	0.0029	−0.0013	−0.0037	99.6136	33.5343

### Randomness Detection

Randomness detection examines whether the detected sequence demonstrates the characteristics of the random sequence, using the techniques of probability statistics. The most authoritative package for the randomness test is the Special Publication 800-22, provided by the National Institute of Standards and Technology (NIST) of the United States (Khawaja and Khan, 2019). This test package uses the P-value returned for different aspects of the evaluation process for making the judgment. Only when each P-value is greater than 0.01, the test sequence is recognized as a random sequence. In this study, the randomness of the encrypted image of “MRI-Brain” is examined here as an example, and the results are shown in Table 17. The results of all the test items in Table 17 are “success,” which proves that the encrypted image obtained by using the proposed algorithm has good randomness.

**TABLE 17:** NIST randomness test of encrypted images.

**Test**	***P***-**values**	**Results**
Frequency	0.139830	Success
Block frequency	0.747300	Success
Rank	0.944274	Success
Run (*M* = 10,000)	0.240022	Success
long runs of ones	0.937168	Success
Linear complexity	0.618749	Success
Overlapping templates	0.446549	Success
Non-overlapping templates	all *P*-value > 0.01	Success
FFT	0.967619	Success
Approximate entropy	0.801709	Success
Universal	0.507906	Success
Serial P values 1	0.275633	Success
Serial P values 2	0.295743	Success
Cumulative sums forward	0.168961	Success
Cumulative sums reverse	0.075333	Success
Random excursions	all *P*-value > 0.01	Success
Random excursions variant	all *P*-value > 0.01	Success

### Efficiency of the Proposed Algorithm

The efficiency of the algorithm is determined by the time expense of the algorithm. The time cost of the proposed algorithm isO(41MN + 5M + 20N). Table 18 lists the results comparison with other algorithms. From Table 18, it is concluded that the encryption efficiency of the proposed algorithm is higher than other ones in the literature.

**TABLE 18:** Comparison of efficiency.

**Algorithm**	**Complexity**
Proposed	O(41MN + 5M + 20N)
Hua and Zhou, 2017	O(108MN + 72L4)
Sun, 2018	O(579MN)
Belazi et al., 2019	O(124MN)

## Conclusion

For medical images with large storage space and high pixel redundancy, the encryption effect, security and efficiency of encryption algorithm should have higher standards. The proposed algorithm combines the SHA-256 and the hamming distances to obtain the keys, uses the excellent FHCOC system to realize the best DNA dynamic coding, to generate the DNA dynamic chains of different lengths, to carry out dynamic deletion operation and dynamic transposition operation of DNA chains. Test results show that the full diffusion of bases causes the pixels of medical images to be completely disorganized; the efficiency is higher and can resist all common attacks. Of course, the proposed algorithm is not only suitable for medical image encryption, but also suitable for other image encryption scenarios. For future research, the proposed algorithm can be applied to large storage space and parallelism of DNA computing for the protection of medical images.

## Data Availability Statement

The original contributions presented in the study are included in the article/supplementary material, further inquiries can be directed to the corresponding author.

## Author Contributions

XLX and CJZ: conceptualization. XLX: methodology. HYJ and DSZ: formal analysis. XLX: investigation and writing – original draft preparation. HYJ and CJZ: writing – review and editing. DSZ and CJZ: funding acquisition. All authors have read and agreed to the published version of the manuscript.

## Conflict of Interest

The authors declare that the research was conducted in the absence of any commercial or financial relationships that could be construed as a potential conflict of interest.

## Footnotes

1 https://medpix.nlm.nih
